# “There is Nowhere Else That I’d Rather be Than with Them”: Parents’ Positive Experiences Parenting Autistic Children

**DOI:** 10.1177/23969415251357222

**Published:** 2025-07-06

**Authors:** Melanie Heyworth, Catherine McMahon, Diana Weiting Tan, Elizabeth Pellicano

**Affiliations:** 1 Macquarie University, Sydney, Australia; 2 Reframing Autism, Sydney, Australia; 3 Lifespan Health & Wellbeing Research Centre, Macquarie University, Sydney, Australia; 4 University of Western Australia, Perth, Australia; 5 University College London, London, UK

**Keywords:** Autism, parenting, joy, neurotype

## Abstract

**Background and Aims:**

A significant body of research focuses on the negative outcomes of parenting Autistic children, particularly when parents identify as non-Autistic. Less attention has been paid to the experiences of Autistic parents of Autistic children, and even less to the positive or fulfilling elements of parenting Autistic children, regardless of parent neurotype. This study therefore asked: What do parents value about their Autistic children, and what characterizes parents’ positive relationships with their Autistic children?

**Methods:**

Here, 40 Autistic and 40 non-Autistic parents completed semistructured interviews to understand better their positive experiences of parenting Autistic children. We analyzed participant responses using reflexive thematic analysis, using an inductive approach.

**Results:**

We identified five themes: parenting is both challenging and enjoyable and “there's the good and the bad and the highs and the lows” (theme 1); parents value time with their Autistic children and “there is nowhere else that I’d rather be than with them” (theme 2); parents value their Autistic child's personality and “just love watching her be who she is” (theme 3); parenting Autistic children is fulfilling and “I know I must be doing something right” (theme 4); and collaboration, learning, and acceptance are key to parenting fulfillment so that “I wouldn’t have it any other way” (theme 5). Overall, parents told us that parenting could be complex and challenging, and that they had good and bad days. But many parents felt happy to parent their Autistic child, and they enjoyed laughing and doing things together with their child. Parents often really liked their Autistic child, who they thought was caring, funny, and interesting, and they described enjoying their child's company, speaking about the qualities and characteristics they valued in their Autistic child. Some parents felt happy when their child needed them. They liked it when they could help their child and make their child feel safe. This made them feel like they were doing a good job as parent. Parents discussed the personal positive impact of experiencing feelings of self-efficacy and the role of Autistic children in their personal growth. Finally, parents thought that it was important to accept their child and that their life would be different than what they imagined. They reflected on the role of acceptance and flexibility in their experiences of joy and fulfillment.

**Conclusions:**

We show that both Autistic and non-Autistic parents of Autistic children have positive, joyful, and valuable parenting experiences. Our findings have far-reaching implications, including how researchers and practitioners conceptualize parenting Autistic children, and the ways in which parents can be supported to foster such experiences.

## Introduction

As autism diagnoses increase globally (Talantseva et al., 2023; Zeidan et al., 2022), research focused on the wellbeing of Autistic^1^ people (Ayres et al., 2018; Cooper et al., 2024; Najeeb & Quadt, 2024; Pellicano et al., 2022), and their families and carers (Tint & Weiss, 2016; Turnage & Conner, 2022; Vasilopoulou & Nisbet, 2016), is expanding. A holistic focus on Autistic people and their families should enhance our understanding of how families support their Autistic children and are in turn supported in their parenting journey. However, research into the experiences of parents of Autistic children all-too-often focuses on parenting challenges and difficulties, the detrimental impacts of parenting, and ways to ameliorate these (e.g., Saccà et al., 2019; see Meleady et al., 2020), with little attention to parenting benefits or fulfillment. This reflects a more general focus on the costs and impositions of parenting even in the broader literature, in which many studies attend to the tangible, immediate, and detrimental effects of becoming a parent (Luhmann et al., 2012; Nomaguchi & Milkie, 2020), and only rarely explore the abstract experiences of parenting joy and stimulation (Hoffman & Hoffman, 1973; McMahon, 2022; Senior, 2014). Nevertheless, as autism researchers move away from a deficits-based, biomedical understanding of what it means to be Autistic or to care for an Autistic person, toward a focus on flourishing and wellbeing (Dantas et al., 2025; Pellicano & Heyworth, 2023), understanding the positive and fulfilling parts of parenting Autistic children is vital both to identify the mechanisms by which parents might foster parenting fulfillment, and to reorient discussions around parenting toward thriving, rather than surviving.

Currently, the literature paints a particularly negative picture of the experiences of parents of Autistic children. Primarily quantitative studies have reported reduced overall wellbeing (Blacher & McIntyre, 2006), clinically significant stress (Cachia et al., 2016; Enea & Rusu, 2020; Yorke et al., 2018), elevated depression and anxiety (Lam et al., 2024; Megreya et al., 2020), poor physical health outcomes (e.g., Seymour et al., 2013), reduced family quality of life (Hsiao, 2018) and poor family functioning (Temelturk et al., 2021), low individual quality of life (Turnage & Conner, 2022; Vasilopoulou & Nisbet, 2016), reduced parental self-efficacy (Farmad et al., 2024), and social isolation (Davy et al., 2022; Myers et al., 2009). While current studies have hyper focused on the negative experiences of parenting Autistic children, it is clear that parents of Autistic children can face significant challenges, and it is specifically parents of Autistic children who experience these negative impacts at levels above those felt by parents of typically developing (TD) children and children with other disabilities (Curley et al., 2023; McStay et al., 2014). In addition to affecting parental outcomes, Temelturk et al. (2021) suggest that autism independently predicts disordered relationships between a parent and their Autistic child. Although Autistic children can form strong, loving, and secure attachment bonds with their parents (Dugdale et al., 2021; Teague et al., 2018), research indicates that secure attachment relationships are less common in the Autistic population. Indeed, parent–Autistic child relationships may be characterized by less sensitivity, less flexibility, and less attuned bidirectionality than TD child–parent dyads (Apicella et al., 2013; Schore, 2014; Sivaratnam et al., 2015; Teague et al., 2018).

Traditionally, a child's Autistic emotional, sensory, and behavioral profiles have been seen as key determinants of such negative outcomes (Enea & Rusu, 2020; Tarver et al., 2019; Yorke et al., 2018). On this account, the so-called “severity” of an Autistic child's “challenging” or “problem” behaviors (e.g., perceived defiance and avoidance), and of social reciprocity “impairments,” are correlated with higher parenting stress levels, parental mental illness, and parent–child insecure attachments (Barroso et al., 2018; Ghanouni & Hood, 2021; McStay et al., 2014; Teague et al., 2018; Temelturk et al., 2021; Yorke et al., 2018). In the extant literature, this causal relationship is often presumed to be unidirectional, with the extent and nature of Autistic children's “behavior problems” being directly related to increased parental stress (Hayes & Watson, 2013), although some studies have shown that parenting practices (e.g., Suvana et al., 2024), styles (e.g., Clauser et al., 2020), and profiles (e.g., Greenlee et al., 2023) can affect Autistic children's behaviors and outcomes. Additionally, factors such as financial resources (e.g., Trentacosta et al., 2018), developmental knowledge (e.g., Campbell et al., 2019), and educational level (e.g., Kavaliotis, 2017) have been shown to affect parents’ experiences, which also vary depending on the age of the child. Importantly, much of the literature evidencing this narrative—and the negative outcomes underpinning it—is quantitative, and less attention has been paid to the experiential and interpersonal complexity and nuance of child–parent relationships, which might be more fully captured by qualitative approaches (Braun & Clarke, 2022).

Some research has examined how parenting challenges can be mitigated (e.g., Ni’matuzahroh et al., 2021). For example, parental acceptance has been suggested to provide a protective effect on the parent–child relationship and on parent wellbeing (Da Paz et al., 2018; Di Renzo et al., 2020; Naicker et al., 2022). Parental self-compassion has been shown to be positively associated with life satisfaction and negatively associated with parental depression and stress (Neff & Faso, 2015), as well as a protective factor against affiliate stigma (Torbet et al., 2019). Manicacci et al. (2019) have also demonstrated that certain parental—rather than child—factors, such as emotional intelligence, resilience, and coping strategies, protect against the risk of increased parental distress when parenting an Autistic child. Nevertheless, the possibility that a *child's* Autistic profile and unique traits and personality might *positively* affect parents’ experiences has rarely been examined, and studies focusing specifically on the correlation between parenting an Autistic child and parenting *joy* are largely absent.

Another limitation to our current understanding is the role of parent neurotype in parenting experiences. On the one hand, when compared to their non-Autistic peers, the experiences of Autistic parents are relatively unknown (Thom-Jones et al., 2024). This means that we do not know whether those factors impacting non-Autistic parents’ experiences (such as parenting style, profile, financial circumstances, and developmental knowledge) and strategies promoting non-Autistic parental wellbeing (like acceptance and self-compassion), apply to Autistic parents as well. On the other hand, most extant studies that are not explicitly focused on Autistic parents do not report parent neurotype at all, so it is impossible to know the neurotype of most studies’ participants. Such lack of detail means that we do not know whether neurotype is a variable in parents’ experiences—negative or positive—of parenting an Autistic child.

Notably, studies that have attempted to present a more nuanced picture of parents’ experiences (e.g., Corcoran et al., 2015; Meleady et al., 2020; Myers et al., 2009; Teague et al., 2018) have focused specifically on *Autistic* maternal experiences (Donovan et al., 2023; Dugdale et al., 2021; Heyworth et al., 2023; Libster et al., 2024; Pohl et al., 2020; Smit & Hopper, 2023; Sutcliffe-Khan et al., 2024; Thom-Jones et al., 2024). These studies show that, despite challenges, Autistic parents can find loving, joyful, rewarding, and enjoyable elements or moments when parenting their Autistic children. Nevertheless, what is largely missing from the literature are studies focused *directly* on the positive aspects of parenting Autistic children (see Meleady et al., 2020; cf. Chau & Furness, 2023), from Autistic *and* non-Autistic perspectives. An in-depth, qualitative study of parental experiences which attends to parent neurotype *and* positive experiences is thus warranted.

### The Current Study

In the current study, we sought to examine moments of parenting joy and connection for both Autistic and non-Autistic parents of Autistic children, to consider the specific ways in which parents experience their parenting journey positively. To do so, we drew on data collected through semistructured interviews with Autistic and non-Autistic parents of Autistic children for a broader study on their experiences of parenting their Autistic children. During that broader study, it became apparent that both Autistic and non-Autistic parents often experienced deep bonds with their Autistic children, and spoke of parenting joy in similar ways. To understand these experiences in greater depth, we therefore posed the following research questions: What do parents value about their Autistic children, and what characterizes parents’ positive relationships with their Autistic children? This study thus contributes to our knowledge by explicitly examining both Autistic and non-Autistic parents’ experiences of parenting joy and fulfillment.

## Method

Eighty semistructured interviews from Autistic (n = 40) and non-Autistic (n = 40) parents of Autistic children were analyzed qualitatively, utilizing reflexive thematic analysis (Braun & Clarke, 2022), within an inductive, essentialist framework.

### Participants

Participants were recruited through convenience and snowball sampling, via social media and through the first author's community networks. Eligible participants were required to be from Australia; English speaking; willing to communicate about their parenting experiences; have at least one primary-school aged child (6- to 12-year-old) with a clinical autism diagnosis, and—for non-Autistic parents—also have at least one non-Autistic child of primary-school age. This final criterion was to address research questions not applicable to the current study. We chose to speak to parents of children in middle childhood, so that we could assess parenting experiences that were not characterized by the complexities of early childhood (e.g., Sandbank et al., 2023), or the additional challenges often associated with adolescence (e.g., Tesfaye et al., 2022).

Forty non-Autistic and 40 Autistic parents (either formally diagnosed or self-identified) met eligibility criteria. As is becoming a more regular practice in autism science (e.g., Davies et al., 2024), we chose to include self-identified Autistic parents in our sample to acknowledge the significant barriers (including lengthy waiting lists and prohibitive costs) to adult autism diagnosis (Ardeleanu et al., 2024). Of the Autistic participants, 29 (72%) were formally diagnosed and 11 (28%) self-identified as Autistic; all were identified in adulthood (*M* = 39.59 years, SD = 5.48).

Participants (n = 80) were between 26 and 55 years (*M* = 41.99 years, SD = 5.19) and were mostly women (87%) (see Table 1 for breakdowns in Autistic and non-Autistic participant demographics; see Supplemental materials T1 for full demographics). While participants had varied cultural heritage, they predominantly identified as Australian (79%), including three (4%) Aboriginal and/or Torres Strait Islander Peoples. Most were well-educated (71% were university graduates), over half were employed full- or part-time (58%), and most resided in metropolitan areas (76%). Regardless of neurotype, many participants reported experiencing at least one mental or physical health condition, primarily attention deficit hyperactivity disorder (ADHD; 32%) and anxiety (48%). Seventy participants (88%) parented their child alongside a partner living in their home, either as a co-parent or the primary caregiver; 10 (12%) participants identified as solo (single) parents.

**Table 1.** Summary of Participant Characteristics.

Participant Characteristics	Parents of Autistic Children (n = 80)		
	All Parents (n = 80)	Autistic Parents (n = 40)^a^	Non-Autistic Parents (n = 40)^b^
	Mean (SD), Range, or N (%)^c^		
Age (years)	42 (5.2), 26.5–55.3	42.20 (4.97), 31.92–55.25	41.79 (5.39), 26.5–52.17
Age at autism diagnosis (years)^d^	NA	39.59 (5.48), 29–54	NA
Interview duration (min)^e^	85 (21.1), 43–195	80.03 (17.64), 43–120	89.92 (22.91), 52–195
Gender			
Woman	70 (87.5%)	35 (87.5%)	35 (87.5%)
Man	7 (8.8%)	2 (5%)	5 (12.5%)
Nonbinary	3 (3.8%)	3 (7.5%)	0 (0%)
Transgender	3 (3.8%)	3 (7.5%)	0 (0%)
Participants’ country of birth			
Australia	63 (78.8%)	30 (75%)	33 (82.5%)
Outside Australia	17 (21.3%)	10 (25%)	7 (17.5%)
Aboriginal or Torres strait Islander	3 (3.75%)	2 (5%)	1 (2.5%)
Parenting arrangements			
Co-parenting	40 (50%)	19 (47.5%)	21 (52.5%)
Primary caregiver	30 (37.5%)	15 (37.5%)	15 (37.5%)
Solo parenting	10 (12.5)	6 (15%)	4 (10%)
Educational attainment			
High school	3 (3.75%)	2 (5%)	1 (2.5%)
Trade/TAFE qualification	18 (22.5%)	10 (25%)	8 (20%)
University qualification	57 (71.25%)	27 (67.5%)	30 (75%)
Other	2 (2.5%)	1 (2.5%)	1 (2.5%)
Co-occurring conditions			
ADHD	25 (31.5%)	19 (47.5%)	6 (15%)
Anxiety	38 (47.5%)	23 (57.5%)	15 (37.5%)
Depression	28 (35%)	17 (42.5%)	11 (27.5%)
Other	47 (58.8%)	34 (85%)	13 (32.5%)
Undisclosed	7 (8.75%)	2 (5%)	5 (12.5%)

a Two Autistic parents chose “I am questioning my Autistic identity” in the demographic survey; since these participants consistently identified themselves as Autistic in the interview and in email correspondence, they have been included in the Autistic parent cohort.

b Four non-Autistic parents chose “I am questioning my Autistic identity” in the demographic survey; since these participants consistently identified themselves as non-Autistic in the interview and in email correspondence, they have been included in the non-Autistic parent cohort.

c Data are mean (SD; range) or n (%). Percentages may not sum to 100% due to rounding issues.

d Age at formal diagnosis, or age at which participants began to self-identify.

e Five participants (three Autistic and two non-Autistic) chose to complete their interviews in writing.

ADHD=attention-deficit hyperactivity disorder; NA = not applicable.

Participants described their relationship with 87 Autistic children, with seven (28%) Autistic participants speaking about more than one Autistic child. Children's ages ranged from 6 to 12 years (*M* = 10.03 years, SD = 1.67) (Table 2; see Supplemental materials T2 for full demographics), with children's age at autism diagnosis ranging from 1 to 11 years old (*M* = 6.38 years, SD = 2.62). Participants spoke about 35 (40%) girls, including two transgender girls, 50 (57%) boys, and two (2%) nonbinary children. Most children used speech to communicate (95%) and were at mainstream school with (41%) or without (23%) additional support. Thirteen children (15%) were homeschooled. Most children (85%) had at least one co-occurring condition, most commonly ADHD (63%) and anxiety (54%). Five children (6%) had co-occurring intellectual disability. The majority (94%) were supported by a National Disability Insurance Scheme (NDIS)^2^ funding package.

**Table 2.** Summary of Autistic Child Characteristics, as Reported by Parents.

Child Characteristics	All Parent Participants (n = 80)	Autistic Parent Participants (n = 40)	Non-Autistic Parent participants (n = 40)
	Mean (SD), Range, or N (%)^a^		
Total number of Autistic children	n = 87	n = 47	n = 40
Age (years)	10 (1.7), 6.3–12.9	10.1 (1.7), 7.3–12.8	10 (1.8), 6.3–12.9
Diagnosis			
Autism spectrum disorder	85 (97.7%)	46 (97.9%)	39 (97.5%)
Autistic disorder	1 (1.2%)	1 (2.1%)	0 (0%)
Asperger's syndrome	1 (1.2%)	0 (0%)	1 (2.5%)
Age at autism diagnosis (years)	6.38 (2.6), 1.1–11.8	6.22 (2.5), 1.1–11.3	6.56 (2.8), 2–11.8
Gender			
Girl	35 (40.2%)	20 (42.6%)	15 (37.5%)
Boy	50 (57.5%)	25 (53.2%)	25 (62.5%)
Nonbinary	2 (2.3%)	2 (4.3%)	0 (0%)
Communication			
Speaking	83 (95.4%)^b^	46 (97.9%)^c^	37 (92.5%)^d^
Nonspeaking	4 (4.6%)	1 (2.1%)	3 (7.5%)
Current educational setting			
Specialist school or unit	10 (11.5%)	4 (8.6%)	6 (15%)
Mainstream with support	36 (41.4%)	17 (36.2%)	19 (47.5%)
Mainstream without support	20 (23%)	9 (19.2%)	11 (27.5%)
Other	21 (24.1%)	17 (36.2%)	4 (10%)
Co-occurring conditions			
ADHD	55 (63.2%)	29 (61.7%)	26 (65%)
Anxiety	47 (54%)	27 (57.5%)	20 (50%)
Intellectual Disability	5 (5.8%)	2 (4.3%)	3 (7.5%)
None	13 (14.9%)	7 (14.9%)	6 (15%)
Other	51 (58.6%)	32 (68.1%)	19 (47.5%)
NDIS plan^e^			
No	5 (5.8%)	3 (6.4%)	2 (5%)
Yes	81 (94.2%)	44 (93.6%)	37 (92.5%)
Pending application	1 (1.2%)	0 (0%)	1 (2.5%)

a Data are mean (SD; range) or n (%). Percentages may not sum to 100% due to rounding issues.

b Of these, four parents reported that their child had a significant language delay (n = 4, 5%).

c Of these, two parents reported that their child had a significant language delay (n = 2, 4%).

d Of these, two parents reported that their child had a significant language delay (n = 2, 5%).

e NDIS plan listing autism as the primary disability.

ADHD=attention-deficit hyperactivity disorder; NA = not applicable; NDIS=National Disability Insurance Scheme.

### Procedure

Ethical approval for the study was received from the Human Research Ethics Committee at Macquarie University (Project ID 11445). All participants gave written, informed consent prior to participation.

A semistructured interview was developed by MH in collaboration with CM and EP and an advisory group of three Autistic and non-Autistic parents of Autistic children with varying support needs. The advisory group was established at the outset of the broader project and helped to shape the interview trajectory and questions; their input ensured that the interview was respectful of parents’ varying experiences and facilitated participant-led exploration of those experiences (see Supplemental materials S3 for details). The interview's purpose was to understand broadly the breadth of experiences of parents of Autistic children, and any differences they perceived between parenting their Autistic and non-Autistic children, if applicable.

MH conducted all interviews between September 2022 and October 2023. Efforts were made to meet the communication, processing, and sensory needs of participants where possible. Seventy-one participants (89%) chose to do their interviews via Zoom. Four (5%) chose to be interviewed by telephone, and five (6%) in writing, through an individualized Word document on which they contributed their answers. Participants were first asked to complete a Five-Minute Speech Sample (FMSS; Magaña-Amato, 2020), and then to answer questions about their parenting experiences in a semistructured interview (see data analysis below; Supplemental materials S3).

The FMSS requires parents to speak uninterrupted for 5 min about what kind of a person their child is, and how the parent and child get along together. The FMSS is validated to measure “expressed emotion,” or the levels of criticism, hostility, and emotional over involvement spontaneously expressed by a caregiver about the person to whom they provide care (Magaña et al., 1985). The FMSS was included here to answer research questions from the broader study about expressed emotion that do not pertain to this specific study. The FMSS is usually delivered using speech; a version was thus adapted for those participants who chose to complete their interview in writing (see Supplemental materials S3).

The semistructured interview then invited parents to reflect on their broad parenting experiences (see Supplemental materials S3). A subset of these interview questions asked parents about the fulfilling and joyful parts of parenting an Autistic child, including what activities parents engaged in with their Autistic child, their attachment or connection, and their experiences of parenting self-efficacy. Thematic analyses of other interview questions, pertaining to other parenting experiences, are reported on separately elsewhere, including on the impact of the double-empathy problem (Milton, 2012) on cross-neurotype parent–child dyads, and on Autistic parents’ self-perceptions of their parenting challenges and strengths (see, e.g., Heyworth et al., 2025a; 2025b).

All participants were emailed questions in advance of the interview, to help them prepare and to accommodate different processing needs, as well as ways to request a break or stop the interview. Given the current literature on the experiences of parents of Autistic children, we anticipated that some participants could find discussing their experiences distressing; information on support services was thus sent immediately after the interview. Further ethical considerations pertaining to this study are discussed in Heyworth (2024).

Interviews conducted over Zoom or telephone (n = 75) ranged from 43 to 195 min (*M* = 85, SD = 21.07), and were recorded with participants’ permission. Interviews were transcribed verbatim, and the de-identified transcripts were emailed to participants to check and edit if they desired, to establish safety for participants in sharing their stories. Three (4%) participants provided additional information after their interviews.

Participants were also asked to provide detailed demographic data following the interview, which were collected and managed using REDCap electronic data capture tools hosted at Macquarie University (see Heyworth, 2024, for discussion of the methodological considerations underpinning the decision to collect demographic data after rather than before the interview). They were paid an AUD50 gift voucher for their time and contributions.

### Data Analysis

We followed Braun and Clarke's (2022) six-phase process for reflexive thematic analysis, adopting an inductive, essentialist framework. While in the broader study, the FMSS data were coded using the FMSS coding schema (Magaña-Amato, 2020) and its autism-specific counterpart (Daly & Benson, 2008), for the purposes of this study, the FMSS data were also analyzed qualitatively alongside the other interview data. Usually, FMSS are coded to capture the nature and number of a parent's comments, according to strict criteria. Coding FMSS qualitatively allowed us to focus specifically on the content of parents’ speech samples, including their spontaneous descriptions of their relationships with their child and their representations of their child's qualities, as these pertain to this study's research questions (see Greenlee et al., 2024; Owens et al., 2022). For this analysis then, the FMSS has been treated simply as another interview question.

To begin, MH immersed herself in the data, listening to the audio of each interview and then reading each interview transcript twice. DT and EP read a selection of transcripts from both Autistic and non-Autistic parents to familiarize themselves with a sample of the data. MH then worked systematically through the data, applying code labels to capture relevant and meaningful concepts. Initially, MH coded interviews separately according to parent neurotype (Autistic and non-Autistic), managed in NVivo Version 14. Given the significant overlap in codes and potential themes, however, the groups were combined for subsequent analysis. All authors discussed the codes repeatedly, with MH making necessary changes to better capture the semantic meanings in the dataset. MH then grouped codes together to identify candidate themes and subthemes, which she discussed with DT and EP. MH developed the themes by generating a thematic map and collating data under relevant themes and subthemes, which was reviewed by DT and EP and subsequently refined by MH. A draft analysis was shared and discussed with CM, DT, and EP and revised based on discussions.

### Positionality

MH is an Autistic researcher, parent to Autistic children, and a systemic advocate who works for an Autistic-led charity. CM, DT, and EP are non-Autistic autism researchers with a deep commitment to participatory, inclusive research. Across all phases, MH kept a reflexive journal to record her thoughts and reactions to participants’ responses, and these were discussed with DT and EP during team meetings. Such reflexivity was particularly important given MH's status as an “insider researcher” (Braun & Clarke, 2022), to help both manage the emotional labor involved in analysis, as well as to record and recognize the ways in which her positioning and assumptions shaped the analysis. Analysis was thus both iterative and reflexive.

### Community Involvement

This study was a collaboration between Autistic and non-Autistic researchers, and was conceived of by MH, an Autistic researcher and parent of Autistic children. The broader study, from which this analysis is drawn, was guided by an advisory group of three Autistic and non-Autistic parents of Autistic children, with diverse support needs. The advisory group members offered insight across the life of the project, including into the design of the study, the interview protocol, all study materials, data analysis, and reviewing this article. Advisory group members were not participants in the study. Four parent participants (two non-Autistic and two Autistic) reviewed a draft of this manuscript prior to publication for sense checking.

## Results

**Figure 1.**
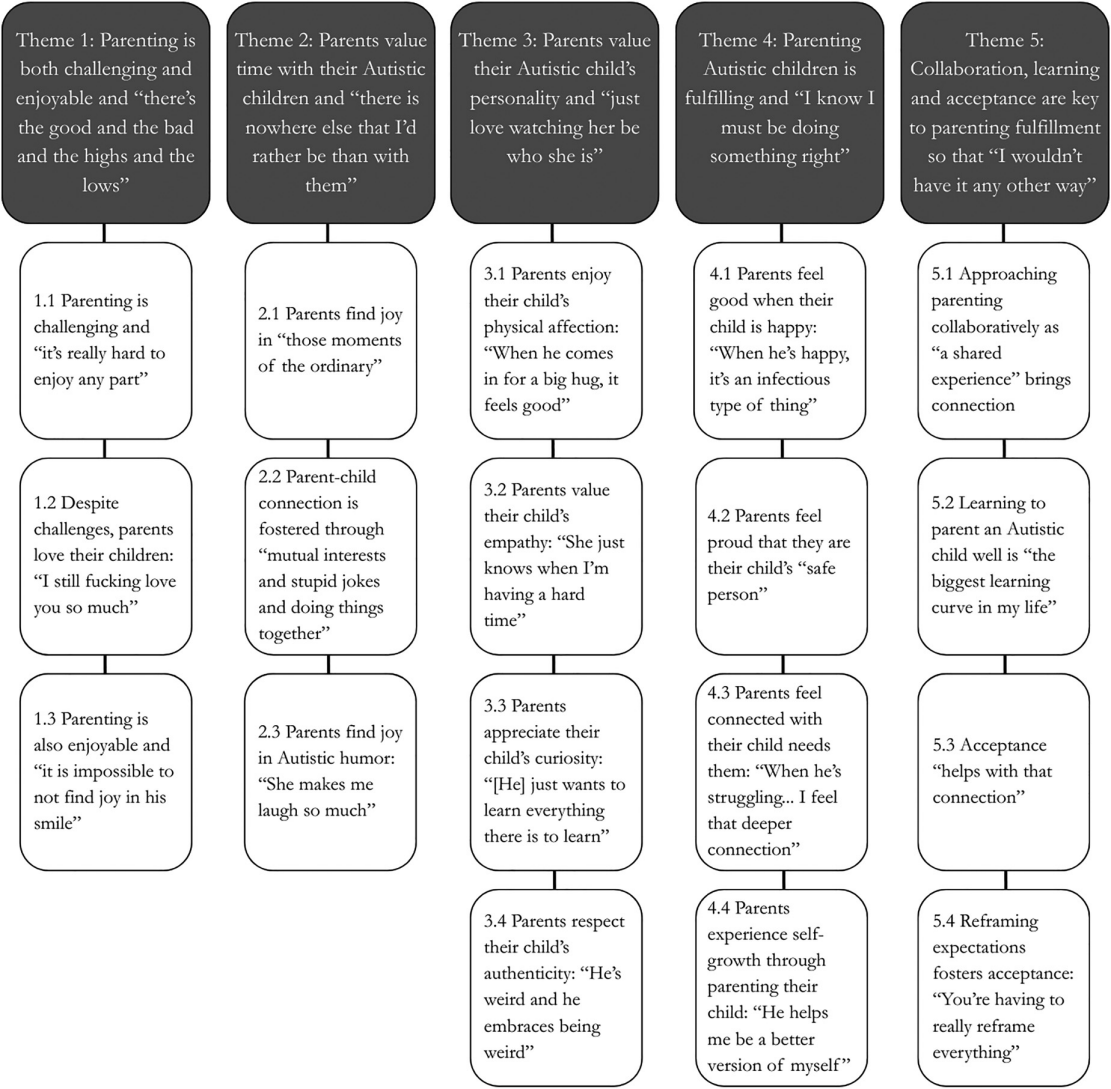
Parents’ Positive Experiences of Parenting Their Autistic Child: Themes and Subthemes.

We developed five themes pertaining to parents’ positive experiences of parenting their Autistic child (Figure 1). We present these themes and subthemes below in bold and italics, respectively, alongside illustrative quotations, attributed to participant by anonymized IDs. Autistic parents’ responses are indicated by the prefix “AU_”, and non-Autistic parents’ by “NA_”.

### Theme 1: Parenting is Both Challenging and Enjoyable and “There's the Good and the Bad and the Highs and the Lows” (NA_OVSC)

Most parents, regardless of their neurotype, found it difficult to categorize their relationships with their Autistic children as wholly positive or negative, and parent–child relationships were conceived of as complex and varying, depending on time and context. Participants thus spoke of their relationship with their Autistic child as nuanced: “it's like a glass plinth, it's fragile, but it's solid as well” (NA_5N7S).

#### Subtheme 1.1 Parenting is Challenging and “It's Really Hard to Enjoy Any Part” (AU_8HYZ)

Parents acknowledged that parenting could be challenging. They explained that their expectations for what parenting would be like had often been unmet. Some parents expressed “just sadness, it's not how I wanted it to be” (NA_3WIO). As one mother put it, she “didn’t really sign up for all of this” (NA_1ZFU). Another highlighted that the mismatch between her expectations of life as a parent and her lived reality was “the really hard part, cognitively and emotionally, to just come to terms with” (NA_3LTP). While some parents grew to embrace this change in expectations (see subtheme 5.3), others emphasized that the day-to-day challenges of parenting their Autistic child were “hard” (AU_GKIQ), “a huge struggle for me” (AU_OOFF), “frustrating” (NA_2QIW), “fraught” (NA_80YU), and “difficult” (NA_DTG1).

For some parents, the everyday challenges of parenting were overwhelming, which led to them to report that “it's really hard to enjoy any part” (AU_8HYZ) and “I wish I’d never bloody go on, wish my whole course of my life had changed” (NA_3WIO). As one parent described, “it's slog non-stop” (AU_SAUF), and another said that “everything always just felt negative” (NA_ZYOF). When asked if some parts of their parenting were joyful or fulfilling, some parents simply replied “no,” “not really,” or “not many.” One parent simply did not expect to find parenting joyful, asking “Is parenting supposed to be joyful? I mean it can be pleasant but it's not like I’m expecting some joy… it's not about me anymore, it's just about the kids” (NA_HYI4).

#### Subtheme 1.2 Despite Challenges, Parents Love Their Children: “I Still Fucking Love You so Much” (NA_XWZP)

Yet, despite the struggles, parents nevertheless described profound, loving relationships with their children: “I still fucking love you so much” (NA_XWZP). Some parents described their children as their “soulmate” (AU_5YKG), “buddy” (NA_VIMZ), or “best friends” (AU_VT9X), with whom they shared a “soul bond” (NA_6F6X). Even when parents felt overwhelmed by the enormity of the challenges facing them (see subtheme 1.1), they still expressed deep love for their child: “I would not take her back… because I absolutely love her” (NA_3WIO). Most parents, then, understood their relationships with their Autistic child “like a complicated love” (AU_TGUX), with challenges and love both characterizing their experiences.

#### Subtheme 1.3 Parenting is also Enjoyable and “It is Impossible to not Find Joy in His Smile” (NA_BG1A)

Many parents found some joy, enjoyment or fulfillment in their parenting experience, or in their connection or relationship with their Autistic child: “It is impossible to not find joy in his smile” (NA_BG1A). Indeed, some parents experienced an all-encompassing joy in parenting their Autistic child, with one parent sharing that parenting is “just pure joy… he makes my day” (AU_6LL4), and another reflecting, “What's not to be happy about really?” (AU_AKPQ). These parents described “enjoy[ing] spending time together” (NA_2QIW) with their Autistic child. They reported feeling “very blessed and lucky that I get to experience him” (AU_6LL4) and “happy together” (AU_64KZ). As one parent put it, “As I say to [Autistic child], ‘you’re my happy every day’, because he's just lovely” (AU_GKIQ).

### Theme 2: Parents Value Time Spent with their Autistic Children and “There is Nowhere Else that I’d Rather be Than With Them” (AU_263B)

#### Subtheme 2.1 Parents Find Joy in “Those Moments of the Ordinary” (AU_PJT0)

For those parents who felt that parenting is an intensely enjoyable and joyful experience, they insisted that joy was not necessarily extraordinary or unexpected in their lives but rather stemmed from “those moments of the ordinary” (AU_PJT0) and the “kind of moments of normality, where you’re like, ah, this is nice” (NA_ALOP). Parents found joy in the “small moments” (NA_87F5) interspersed in everyday life. These parents spoke about “moments of absolute pure joy… and absolute bliss,” even as they acknowledged there also are moments of “everything in between” (AU_OTQY). One parent noted that it was “lovely” to experience the “simple stuff, like watching a movie together” (AU_WOMI). Other parents described more idiosyncratic moments of joy, that were nevertheless, for them, an ordinary part of the everyday. One mother shared that her Autistic child has a “crazy sense of smell and she’ll come up and… just say, ‘mmm, Mummy smell’. It's beautiful” (NA_AS2X). Another shared that “one of my favorite things is when she gets excited, she’ll jump, the Autistic excited jump thing, and I love that” (AU_VB8S). For many of these parents, feeling “joyous all the time” (NA_VJKZ) was the norm.

#### Subtheme 2.2 Parent-Child Connection is Fostered Through “Mutual Interests and Stupid Jokes and Doing Things Together” (AU_HGJ9)

Many parents spoke of finding such “ordinary” joy and fulfillment through connection with their child. Such connection came most easily when there were “mutual interests and stupid jokes and doing things together” (AU_HGJ9)—that is, when there was an organic interest, passion, or pastime shared by both parent and child. Parents described various interests they had in common with their child. One parent explained, “we watch [the TV show] Survivor together, actually, that's our special interest… so we talk a lot about that. And that is really connecting” (AU_CNGI). But parents spoke of diverse shared activities, like reading (“she’ll read her book and I’ll read my book”; NA_FEHX), listening to audiobooks (“we listen to a lot of books together”; AU_PJT0), “shopping and going out” (NA_FVUD), making “apple pies” (NA_L2YJ), doing “art” (AU_OOFF), sharing “our excitement about getting a cat soon” (NA_8Q0V), or just having “a lot of deep conversations” (AU_M9BP). Autistic parents spoke also of connecting with their child through shared neurotype. One Autistic mother of a nonspeaking Autistic child explained her connection with her son through their shared sensory seeking profile: “people go, let's put on some light relaxation music. I’m like, you know what, that shit's boring. I know it is, and he knows it is. Give him some bass. Then we put it up really loud and have some beats” (AU_TGUX). Another explained that “nit-picking how weird NT [neurotypical] people are” (AU_K6KO) was a point of connection with her Autistic child. These shared activities and interests were “how we connect” (AU_IV9M) and a source of “my moments of joy with him” (AU_ZZQH).

Even when parents *did not* organically share in their child's interest, their attempts to be involved in their child's passion facilitated joy and connection. These efforts were seen by some parents as a demonstration of love and as vital to building and maintaining connection with their child: “all her interests, I make sure that like I’m doing them as well… just trying to make sure that I always stay connected and stuff with her” (AU_426V). Or as another put more bluntly:

I have no interest in it, but I follow to make sure she feels like she's being heard… So, if it wasn’t for her, I wouldn’t be interested in those topics but then I know it's something that makes her happy, so you want to make your kids feel heard and happy. (NA_OVSC)

Equally, parents recognized that their child's attempt to share their passion with their parent stemmed from a desire to connect, and parents found fulfillment when “he invites me into his world” (AU_6LL4): “the fact that he wants to share it [with me] is fulfilling for me” (NA_6S9L).

#### Subtheme 2.3 Parents Find Joy in Autistic Humor: “She Makes Me Laugh so Much” (NA_XWZP)

For many parents, sharing their child's humor was a source of joy: “She makes me laugh so much” (NA_XWZP). Contrary to the conventional understanding that Autistic people have “impaired” use and understanding of humor (Mention et al., 2024), many parents spoke not only of their child's “fabulous” (NA_3WIO) or “great sense of humour” (NA_ZGRM), but of their ability to make their parent laugh: “we enjoy laughing together. She loves making me laugh, and then I like joining in with her crazy sense of humour and making her laugh” (NA_2QIW). Another parent described “the dark humour side of” her Autistic son, “evil humour and I am here for it. I love it so much because I’m very the same… the look in his eyes is just pure evil glee” (NA_Y1A1). Many parents, then, described how sharing laughter (“when we’re having a laughing fit together”; AU_9VCO, or “when he has those uncontrollable giggle laughs that he just can’t contain”; NA_7AKR) “brings me really big joy” (AU_426 V). As one parent put it, the “giggle loop” of laughing together was thus important to parents’ enjoyment of their parenting experience (AU_ZZQH).

### Theme 3: Parents Value their Autistic Child's Personality and “I Just Love Watching her be Who She is” (AU_426V)

Some parents found joy in their child's personality. Different parents appreciated different aspects of their Autistic child's identity, but parents described valuing aspects of their child's character that brought them pride, connection, and fulfillment, and observed that “she's just a nice person to be around” (NA_27S1). Thus, even when their Autistic child could frustrate them, or parenting was challenging, most parents identified elements of their Autistic child's personality and/or identity that they were proud of. As one parent noted: “as a person, he just brings me joy for who he is” (AU_RWHQ).

#### Subtheme 3.1 Parents Enjoy Their Child's Physical Affection: “When He Comes in for a Big Hug, It Feels Good” (AU_M9BP)

For some parents, their Autistic child's demonstrations of affection were a source of joy: “when he comes in for a big hug, it feels good” (AU_M9BP). One parent explained that she experienced a deep emotional connection with her Autistic child when “she’ll come and sit on my lap sometimes. And she’ll just want to cuddle” (AU_64KZ). Another parent described her appreciation of her son's physical affection for her: “he gives the best cuddles. So that's where I find the joy” (NA_9CMJ). Parents report that Autistic children were not always affectionate or receptive to their shows of affection, but, when they were, these moments were cherished: “in those moments where we look at each other and where she's kind of showing that she's open to affection, they’re the beautiful moments” (NA_AS2X). Many parents thus found joy in their child's displays of affection, which they took as a sign of a desire to connect. This was particularly true for parents of nonspeaking or minimally speaking children, or children with intellectual disability, who found connection in physical affection: “I love that we’ll sit on the bed and he’ll put his head on me and have a cuddle. He looks for that connection” (NA_IMVT).

Parents acknowledged that their child's overt demonstrations of love and connection might not be “conventional.” One mother described her son's attempt to connect through his passions and “infodumping,” saying “he seeks to connect to others through his love of science” (AU_BY0E). Another parent explained that her ability to recognize her son's sometimes unusual attempts to connect with her were “really special” to her:

It happens on a daily basis, that there's a moment where he reaches out for me, whether it's that he comes and sits right next to me or he offers me popcorn, which is his food. I do recognise the importance for him. (AU_6LL4)

For parents of nonspeaking children or children with language delays, this ability to recognize and respond to a child's desire to connect was pivotal, as one father of a child with language delays and an intellectual disability described:

At no point have I ever been worried that he doesn’t connect, that he doesn’t love me, that he doesn’t want to spend time with me or anything like that. He obviously shows it in a different way than a lot of kids but it's, it's very clear, very clear. (NA_BG1A)

Another parent agreed: “I get that warm feeling from when we’re sitting watching TV together, and he gets his foot, and he puts it on my leg, because that's how he's traditionally shown love” (AU_HNWF).

#### Subtheme 3.2 Parents Value Their Child's Empathy: “She Just Knows When I’m Having a Hard Time” (AU_OTQY)

Many parents spoke specifically of appreciating their Autistic child's empathetic and compassionate approach to life, challenging stereotypes of Autistic empathy. Parents found deep comfort and joy in their experiences of their child's empathy towards them: “She just knows when I’m having a hard time” (AU_OTQY). Parents conceptualized their Autistic child's empathy in various ways. For some, their child's empathy was clear in the way “he reads me better than anyone else” (NA_Q8GB). For others, empathy was equated with emotional intelligence: as one mother observed of her nonspeaking daughter, “there was this deep sort of soul intelligence, kind of, and it also seems to have this emotional element to it… that sort of deep wild soul spirit, which is part of what I love” (NA_6F6X). Other parents again recognized that their Autistic child's empathy was not uncomplicated, but had shared neurotype as its foundation, as one Autistic parent described: “I think there is always an undercurrent of empathy that unites us because we both share challenges and gifts that are different to the majority of people. She says, ‘I love you, Mum, because we’re similar’” (AU_BFDL).

For some parents, their child's compassion and empathy toward others was a source of pride. One parent loved that her Autistic son “is so caring and so compassionate to his little sister” (NA_Y1A1). Such empathetic displays provided joy, as one parent commented of her child: “my joy comes from when I see how kind and thoughtful… and considerate of people and just genuinely warm-hearted [she is]” (NA_87F5). Other parents observed empathy in the way their child connected with and understood them directly. One mother, for example, saw empathy when her child comforted her after she had hurt herself; for her, her child's empathy was a source of parental self-efficacy: “I was like, wow, I’ve done really good with this one” (AU_6LL4). Another felt similarly when her son checked in on her: “so when my child says, ‘how was your day, Mummy? You okay?’, I praise that, because… I love that he's learning to ask that question” (NA_CY2G).

Such joy was amplified when parents appreciated the effort their child was making to connect in moments of empathy, even if—again—such demonstrations of empathy were idiosyncratic or unusual. One parent observed that “when I’m really upset, and he can see that, he will come up an arm's length away and just tap me on the shoulder and that's a huge thing for him” (NA_DTG1). Another observed, “[Autistic child] is not at all a tactile person, and prefers not to touch others, but he will come up to me and touch my hair… to be kind to me. I am so touched when he does this” (AU_LE0X).

#### Subtheme 3.3 Parents Appreciate Their Child's Curiosity: “[He] Just Wants to Learn Everything There is to Learn” (AU_AKPQ)

Parents also valued their child's passion, curiosity, knowledge, enthusiasm and creativity, which often provided a gateway to connection, since “[he] just wants to learn everything there is to learn” (AU_AKPQ). Parents enjoyed their child's “enthusiasm for things… and his understanding and his capacity to learn” (NA_1ZFU), which were a source of pride and joy. Many parents described their child's curiosity, enthusiasm, and ability to learn as “awesome” NA_6S9L) or “amazing” (AU_426V) and a “good thing” (NA_80YU). As one parent noted, she was “impressed and surprised” and “really happy and delighted” at “just how thoughtful he is about stuff that he's interested in” (AU_AKPQ). Frequently, parents identified that their children's knowledge was interesting to them: “He's the most interesting person I know” (NA_9CMJ).

#### Subtheme 3.4 Parents Respect Their Child's Authenticity: “He's Weird and he Embraces Being Weird” (AU_6LL4)

Autistic parents, in particular, valued their child's Autistic authenticity, and their willingness to embrace their Autistic identity and to eschew masking; as one parent described it, “he's weird and he embraces being weird” (AU_6LL4). One Autistic parent talked about her two Autistic daughters being “always genuine,” which was “good” (AU_263B), while another described that “there's just been so much wisdom that's come out of him being himself” (AU_9VCO). For some parents, their child's authenticity and “zero mask[ing]” (AU_6LL4) “brings a lot of joy… in that he's learning that he can just be honest”, which is “a really good quality to have when you’re older” (AU_WPZ6). For others, “[Autistic child]'s authentic way of living is very healing” (AU_LE0X) and reflects a “real integrity… that lights me up” (AU_NHEN). Indeed, it brought some parents immense pride and joy to see their children *as* Autistic individuals. One parent spoke of the “fulfilling parts of parenting” as “watching him grow and thrive now… he knows how to embrace his Autistic truth” (AU_NXCD). One parent expressed deep gratitude that her son is “just so unapologetically who he is” (AU_UULC), which was echoed by others, including non-Autistic parents: “It's those moments of seeing who she and how she feels about life and the world [that] are really beautiful” (NA_AS2X).

### Theme 4: Parenting Autistic Children is Fulfilling and “I Know I Must be Doing Something Right” (AU_BFDL)

#### Subtheme 4.1 Parents Feel Good When Their Child is Happy: “When He's Happy, It's An Infectious Type of Thing” (NA_Q8GB)

When parents saw their children happy, and felt they could understand and help their child to experience happiness, they felt happy too: “when he's happy, it's an infectious type of thing” (NA_Q8GB)*.* Parents explained simply, “I love when she's happy” (AU_64KZ), “our fulfilment comes from his joy” (NA_VIMZ).

One parent recounted the incredible struggles her child had experienced at school, which had resulted in the child experiencing suicidality and post-traumatic stress disorder, which had had a huge impact on “our whole family” (NA_6S9L). While this parent required significant “mental fortitude” to learn what her child needed, and how to provide it for him, it was “seeing [Autistic child] get happier, and blooming at an exponential rate” which fostered, for her, a “sense of achievement and self-efficacy [that] has helped my sense of self-esteem, happiness, and mental health.” Another parent described that since she had recognized and accepted her child's attempts to connect with her and met those attempts with enthusiasm, “I actually realise now, there is enjoyment in there for me as well. And I didn’t really get that before” (NA_Q8GB). One parent, reflecting on her child's and her own unhappiness in the past when compared to their current connected and joyful relationship, observed, “the thing that I have been the most abject failure in, I’m turning around to be probably my proudest achievements” (AU_9VCO).

#### Subtheme 4.2 Parents Feel Proud that They are Their Child's “Safe Person” (AU_BFDL)

Other parents spoke of feeling confident and fulfilled in their parenting in the context of representing “safety” to their Autistic child, and “it makes me feel so special and privileged to be her safe person” (AU_BFDL). Being a child's “safety net and her person” (AU_DBQ6) or “immunity system” (AU_JUWY) could, for some parents, add to their mental load (“it's a lot”; NA_Y1A1) or make them a target for their child's big emotions (“he does lash out towards people who he feels safe with”; NA_GSIL). Yet, parents regularly associated being their child's “safe person” with positive feelings of satisfaction and self-efficacy. One parent described, “it's pretty incredible to feel that way” (NA_Y1A1).

Importantly, some Autistic parents identified that their Autistic child was *their* safe space too, and a person with whom they could be “not perfect” (AU_Y9NF), and they expressed trust in the mutuality of their relationship. For some, “the ability that we can show each other our feelings, that we’re safe in that sense, that we trust and believe in each other strongly” (AU_HGJ9) was the cornerstone of their relationship with their child: “She knows that no matter what, I’m her safe place. And you know, she's my safe place” (AU_HQ88).

#### Subtheme 4.3 Parents Feel Connected When Their Child Needs Them: “When He's Struggling… I Feel That Deeper Connection” (NA_K79N)

For some parents, connection was found not directly in their child's struggle, but rather in their child's need for them as a parent during a period of struggle, and in their own ability to provide safety to their child: “when he's struggling… I feel that deeper connection” (NA_K79N). As one parent observed:

If I lower my voice rather than raise my voice, those are also those things that help to connect. And it sounds funny saying that because it's like the connection is around regulation, but that's also where there's a deep connection. So, he becomes more trusting of me and closer to me. (NA_IMVT)

Another parent felt similarly: “I feel a really deep connection to him when he is wanting to talk to me about stuff that he's finding challenging… And that made me feel really close to him because he was trusting me with something so important” (AU_IV9M). This dynamic meant that parents of Autistic children experienced profound connection with their child, even when—or possibly *especially* when—the relationship or context was challenging or difficult. As one parent reflected on her connection with her Autistic and non-Autistic children:

I really feel bonded to [Autistic child]. I don’t feel that as much with [non-Autistic child]… So, I think [non-Autistic child] is a bit more fickle, she could just attach herself to anybody and be okay… [Autistic child] will only seek that connection from me. (NA_DTG1)

Another parent elaborated, “I think because I have had to look out for [Autistic child], because of the different crises, I’m more in tune [with her]” (NA_6F6X). A parent of a child with complex communication and behavioral needs agreed. For her, parenting was “overwhelming. Stressful. Sometimes almost unbearable” (AU_TGUX), but equally, “when we’re in tune with each other and we’re having a shared moment, it's more special than my other [non-Autistic] child… it's something very beautiful there.” For some, then, having a connection with an Autistic child, is “more memorable and more meaningful than a non-Autistic child” (NA_DTG1).

#### Subtheme 4.4 Parents Experience Self-Growth Through Parenting Their Child: “He Helps me be a Better Version of Myself” (NA_CY2G)

These reflections suggested that parenting an Autistic child—even when that was challenging—presented parents with unique opportunities for connection and parenting satisfaction. For many, parenting an Autistic child also offered opportunities for self-growth, self-reflection, and improved parenting, since “he helps me be a better version of myself” (NA_CY2G). Many parents spoke of parenting as both “a journey but also a privilege” (NA_L2YJ), and a “liberatory” (AU_WOMI) and “rich experience” (AU_NHEN). Some parents felt that “[Autistic child] has very much taught me to be a better parent and I’m grateful for that” (NA_K1C6) since “it's taught me a lot about myself [and] about the world” (NA_ZFOY) and made them “a better person” (NA_87F5): “I wouldn’t be who I am now without her” (NA_FEHX). As one parent explained:

I had no meaning in my life… And then it's just kind of, ‘oh no, I really give a shit about this now’, but it's not because I give a shit about myself. It's more because I give a shit about him. So, he's given me a lot of purpose. (AU_HNWF)

For some parents, this meaning was lifesaving. An Autistic father observed, “I honestly don’t think I would be here today if I didn’t have my kids to be doing it for” (AU_M9BP). Such learning was beyond what “we would have gotten from a neurotypical child” (NA_LXNQ), and the “ultimate wisdom pathway” since “parenting her has absolutely grown me exponentially as a person” (AU_NHEN). One parent explained this process as:

moments where we have been sitting in this deepest connection where I almost feel like he is my teacher. And it just feels so deep and profound that this person has arrived in my life… He's changed me. And I feel so blessed because of that. I really just would never have understood things the way that I understand them, or see things so more meaningfully. (AU_OOFF)

Autistic parents specifically felt that parenting an Autistic child gave them increased insight into their own identity and Autistic experiences, and encouraged them to be authentically Autistic themselves: “he's made me grow and accept myself” (AU_BY0E). Another agreed that “one of the fulfilling things about parenting [Autistic child] is how much he teaches me about how to live authentically” (AU_LE0X). The depth of this process for some was profound: “I think parenting her, has really given me the chance to not only meet an incredible person, but to kind of learn to love parts of myself I really struggled with. And she's taught me to love those things” (AU_ODSH).

### Theme 5. Collaboration, Learning and Acceptance are Key to Parenting Fulfillment So That “I Wouldn’t have it Any Other Way” (NA_8Q0 V)

Unsurprisingly perhaps, most parents did not reflect deeply on the mechanisms or processes that allowed them to find and experience joy and fulfillment in their parenting. Nevertheless, some parents identified that a collaborative approach to parenting, shaped by an acceptance of their Autistic child and a commitment to responsive parenting that adapted to their child's needs, strengthened their relationship with their child and made their parenting journey both easier and more fulfilling. Parents thus explored their own parenting approaches that allowed them to experience joy and connection, and facilitated their fulfillment in parenting their Autistic child.

#### Subtheme 5.1 Approaching Parenting Collaboratively as “A Shared Experience” (AU_Y9NF) Brings Connection

Some parents emphasized their approach to parenting as “a shared experience” (AU_Y9NF). Generally, parents recounted more joy and connectedness when they experienced reciprocity in their relationship with their Autistic child. For some—mostly non-Autistic—parents, feeling unappreciated for “all the thankless jobs you do” (NA_1ZFU) or feeling that their parenting efforts were “often just thrown back in your face” (NA_5N7S) resulted in “feel[ing] down” (NA_3LTP), “very tired, very resentful” (NA_80YU), and that “whatever you do is the wrong thing” (NA_GSIL), “everything goes wrong is my fault” (AU_RW7Q). Nevertheless, regardless of neurotype, many parents characterized their relationship with their child as “co-creating experience” (AU_HGJ9), “very collaborative” (NA_27S1), and “a partnership” (AU_9VCO), in which parent and child “work together” (NA_6S9L); such collaboration resulted in a desire “to grow and learn together” (AU_Y9NF) and in shared “intuition” (AU_ZWO3) that allowed parents to not just extend understanding and love to their child, but indeed to feel these in return. Another parent observed that the “foundations” of her deeply connected relationship with her child was located in such reciprocity, in “that honesty, and respecting each other's interests and curiosity, and wanting to help and support each other” (AU_AKPQ), a sentiment echoed by others who characterized their relationship with their child as bidirectional “trust… It's absolute trust” (NA_6S9L).

Parents thus identified that collaboration through listening and responding to their child's ways of communicating were key to their feelings of self-efficacy. One parent identified strength as “I listen to him and take notice of things” (AU_GKIQ). Another spoke of the importance of “listening to him, but not just kind of auditory listening, but listening in terms of his body language” (NA_L2YJ). Listening and responding to children's needs was thus vital for parents’ own feelings of success: “parenting became a lot easier when you just listen. And you act on what you’re actually seeing and hearing” (AU_VT9X).

#### Subtheme 5.2 Learning to Parent an Autistic Child Well is “The Biggest Learning Curve in My Life” (NA_K1C6)

Importantly, such bidirectionality was not always automatic and took effort to foster, and parenting an [Autistic child] was “*the biggest learning curve in my life*” (NA_K1C6). For some parents, learning to parent their Autistic child, and to appreciate the positives of parenting an Autistic child, required significant learning, humility, vulnerability, and effort. As one father explained, “I’ve struggled many times (especially earlier on) but learnt so much and I’m proud of that” (NA_K1C6). Parents recounted needing to learn, consciously, “how to be a good parent” (NA_8Q0V) to their Autistic child, a process which could be quite “turbulent” and required an openness to “reflection and improve” (NA_8Q0V), but which ultimately made parenting “a lot easier” (AU_RWHQ). Many parents acknowledged this “long journey” (NA_HYI4) was “still a work in progress” (AU_9VCO) and “evolving” (NA_Q8GB) but felt buoyed by the knowledge that parenting gets “better all the time with all we learn” (AY_NXCD) and with “reevaluating my own assumptions” (AU_ODSH). As one parent put it, such learning is “uncomfortable and it's required huge amounts of energy and focus and commitment to unlearning and relearning, but we are all so much better off for it” (AU_WOMI).

#### Subtheme 5.3 Acceptance “Helps with That Connection” (NA_ZFOY)

For some parents, they needed to learn to come to a place of acceptance and understanding of their child's Autistic identity to “help with that connection” (NA_ZFOY) and cultivate a positive, reciprocal relationship with their child. One parent, reflecting on her past decisions to pursue early intensive behavioral therapy with her daughter, identified that learning to accept her child's autism was the key to their present strong bond: “you can’t change it. Embrace” (NA_6F6X). Another parent agreed, describing that, in the past, she had assumed her child's “difficult” behaviors were because he was “deliberately being difficult”, which had undermined their relationship; accepting his Autistic behaviors as “communication” and becoming more “neuroaffirmative” was crucial to this parent's current assessment that “I wouldn’t have it any other way. I love [Autistic child] and the way he is as a person” (NA_8Q0V). With acceptance, then, came understanding and unconditional love, as one parent described: “I think [Autistic child] and my connection is so strong because I am the only person in her life who fully understands her… and loves her unconditionally and she feels this and seeks me out because of it” (AU_BFDL). Such acceptance in turn nurtured attuned, reciprocal connectedness between child and parent.

#### Subtheme 5.4 Reframing Expectations Fosters Acceptance: “You’re Having to Really Reframe Everything” (AU_DBQ6)

For many parents, the journey to acceptance was aided by their ability to be adaptable and adjust their expectations to meet their child's needs—“you’re having to really reframe everything” (AU_DBQ6), rather than dwelling on the idea that their child did not match their expectations. As one parent explained:

No one just says, they’re Autistic, there's nothing wrong with them. But things are going to be different, you just have to face that. And… it's not better or worse, your lives are just, are just different, and you just have accept it. (AU_263B)

Accepting that their expectations would not be met, and responsively changing expectations, was crucial for many parents. One parent remembered her “very authoritarian expectations,” which necessitated a parenting “180” when her family was in crisis: “you’re either forced to continue or you need to change something” (NA_3LTP). For some parents, then, changing expectations to meet a child's needs resulted in improved understanding:

Just a lot of other things are going against the grain of what I thought it should be. For example, like dealing with those anxieties is actually giving her more control, whereas with my Asian background, you don’t give your kid control, that doesn’t happen. (NA_HYI4)

Or, as another expressed, “at first it was challenging to accept almost everything I thought I knew about parenting or my ‘style of parenting’ wasn’t going to work but the ‘retraining’ of my brain has been so beneficial and rewarding” (NA_K1C6). Parenting Autistic children, then, gave parents the permission to “change things” and really “unpack [and]… look at all our expectations” (AU_DBQ6). For some, this included evaluating the neuronormative expectations for parenting, ultimately leading to “an unravelling of my willingness to continue with those expectations and a shedding of my desire to force him into situations that he found painful simply because convention required him to do that” (AU_HGJ9).

## Discussion

For our participants, parenting came with highs and lows, and good and bad times (theme 1). But parents reported finding joy in everyday moments when they connected with their Autistic child over shared interests and humor (theme 2), and through an appreciation of their child's affectionate, empathetic, curious personality, and authentic identity (theme 3). When parents felt good about their parenting—perhaps seeing themselves as a conduit to their child's happiness or safety—they themselves experienced joy, and they appreciated that parenting their Autistic child resulted in personal growth and self-knowledge (theme 4). Some parents identified that working towards accepting their child, collaborating with them, and being adaptable in their parenting might be effortful, but were all ways of maximizing parenting joy and fulfillment (theme 5).

Parenting any child is a complex and nuanced interpersonal endeavor, dependent on many internal and external factors (Pereira, 2021), a reality that has rarely been adequately acknowledged in autism science. Importantly, the experiences of the parents of Autistic children in this study echo the experiences of parents in the general population, for whom parenting is associated with *both* positive and negative aspects and emotions (e.g., Mikolajczak & Roskan, 2018; Negraia & Augustine, 2020). That is, parenting Autistic children—like parenting *any* children—has ups and downs, benefits and challenges, sometimes simultaneously.

In this study, parents often provided rich, balanced, coherent, and positive representations of their Autistic child (Cost et al., 2021; van Bakel & Hall, 2018), and described securely attached relationships, in which parents were sensitive and responsive to their Autistic child's needs. Importantly, the ways in which parents described their Autistic children as affectionate, connected, empathetic, and funny indicate that these Autistic children do not conform to the stereotypes of Autistic communication and sociality (Chevallier et al., 2012), nor to traditional accounts of Autistic “impaired” empathy (Song et al., 2019), humor (Mention et al., 2024), and social-emotional reciprocity (Vivanti & Nuske, 2017). Indeed, parents’ testimonies in this study suggest that established understandings of Autistic social relationships urgently need revision (see Jaswal & Akhtar, 2018; Akhtar & Jaswal, 2020; Pellicano & Heyworth, 2023). Equally, the ways in which participants conceptualized their relationships with their children, and the ways that they valued their children as people—and as *Autistic* people—suggests that the standard accounts of parenting experiences have captured only a limited insight into their fullness.

The prevailing account of parenting Autistic children indicates that a child's behavior and Autistic profile directly impacts parents’ experiences and mental health. Yet this correlation has generally only been examined *negatively*. This study, on the other hand, shows that parents of Autistic children are also impacted *positively* by their Autistic child. In particular, Autistic parents learnt to be authentic through their child's authenticity and “unmasking,” although, as discussed elsewhere, many parents—Autistic and non-Autistic—identified broader personal growth because of their child (e.g., Chau & Furness, 2023; Phelps et al., 2009; Waizbard-Bartov et al., 2019; see Meleady et al., 2020). Moreover, many parents in this study described their relationships with their Autistic children as characterized by mutual physical and emotional connection and affection, even when such connection was intermittent or expressed in nontraditional ways. In other words, this study complements those that focus primarily on *parents’* feelings of deep connection and love for their Autistic child(ren) (Dugdale et al., 2021; Smit & Hopper, 2023; Sutcliffe-Khan et al., 2024) by showing that parents—Autistic and non-Autistic alike—experience a *reciprocated* connection with their Autistic child.

Crucially, participants in this study discussed *how* they experienced such positive relationships with their Autistic children, indicating specific areas that could be targeted in parent education efforts in the future. For example, moments of togetherness, and pride in their Autistic child's personality, ultimately allowed parents to appreciate their own positive influence. Often, how deeply a parent felt they were needed by their child, whether they felt appreciated and loved by their child, and how effectively they felt they could help their child or meet their child's needs, seemed to influence parents’ self-efficacy and enjoyment of parenting. Child happiness, then, sparked parental joy and, in turn, self-confidence in parenting. Thus, parents who felt understood, appreciated, and loved by their child, also often described experiencing joy, connectedness, and self-efficacy.

Given the existing literature, the joy that parents described is perhaps unsurprising for the *Autistic* participants of this study. Previous studies have shown that Autistic parents experience profound love and connection with their Autistic child and experience parenting happiness (e.g., Dugdale et al., 2021; Smit & Hopper, 2023). In these studies, such connection has been accounted for, at least in part, because of shared neurotype (e.g., Crane et al., 2021; Marriott et al., 2022; Winnard et al., 2022), a sentiment echoed by the Autistic parents of this study who understood that parenting an Autistic child “as an Autistic person” means “I come better equipped than some of my neurotypical peers,” is the “best thing” for the child, and that shared neurotype “means that that [parent-child] bond is really, really strong” (see Heyworth et al., 2025b). Certainly, the double empathy problem construct (Milton, 2012)—as well as a plethora of research evidencing the benefits and comparative ease of like-neurotype communication (e.g., Crompton et al., 2020a, 2020b; Watts et al., 2024) and the challenges associated with cross-neurotype communication (e.g., Marocchini & Baldin, 2024)—would suggest that Autistic parents might feel a deeper and easier connection with their Autistic child, than their non-Autistic peers.

This study, however, challenges such an assumption. Separate analysis of the dataset (see Heyworth et al., 2025a for further discussion) suggests that the non-Autistic parents in this study were more likely to find it difficult to understand exactly the motivations and drivers for their Autistic child's behaviors and reactions than the Autistic parents, as we might expect given the double empathy problem, and were also more likely to express feeling unappreciated. Yet here, non-Autistic parents spoke at length about the joy, fulfillment and connection they experienced in parenting their Autistic child in ways that were appreciably similar to Autistic parents’ accounts.

Clearly, not all non-Autistic parents experienced joy and connection with their Autistic child, but neither did every Autistic parent: some participants who were Autistic, and some who were not, felt so overwhelmed by the challenges associated with parenting their Autistic child that their parenting journey was not joyful or connected. Ultimately, for some parents of both neurotypes, the effort of finding moments of joy and fulfillment was unsatisfying. Nevertheless, whilst some elements of parenting fulfillment were unique to Autistic parents—such as learning to be authentic through an Autistic child's authenticity—both groups of parents, Autistic and non-Autistic alike, described moments of intense happiness when they connected with their Autistic child and found joy in their relationships.

The question thus arises: what factors—beyond parent neurotype—facilitate joyful connection?

Previous research has suggested that parental resolution and acceptance of a child's autism diagnosis is an important conduit to parenting satisfaction and positive outcomes (Chau & Furness, 2023; Di Renzo et al., 2020; Jones et al., 2014; Naicker et al., 2022), and the parents in this study felt similarly. Here, many parents linked their joy to an acceptance of their child's Autistic characteristics, behaviors and identity, even when this meant significant adjustment of expectations for their parenting or, indeed, their lives more broadly. For example, parents who connected with their child through shared passions or who invested in their child's passion (even when this was not of organic interest to them) spoke of the joy of such connection. Equally, parents who recognized and responded positively to their child's attempts to connect, even when these were not traditional, understood that such connection brought them joy and fulfillment. This was especially true for parents of children who do not communicate primarily or reliably through speech, as has been demonstrated elsewhere (Jaswal et al., 2020). Importantly, the parents of children who had complex communication needs and/or intellectual disability, spoke not only of challenges, but also of joy and fulfillment, in parenting their Autistic child. These parents spoke of the importance of being aware of their children's idiosyncratic ways of connecting and “accept[ing] the chaos” of parenting. More generally, parents who moved away from “blaming him for being the way he is” to a place of acceptance—that is, who did not identify the child or their Autistic traits as the source of all their parenting challenges—were able to appreciate their child and foster connection with them. Supporting parents to move to a place of acceptance of their child's Autistic identity is thus crucial for their—and their child's—positive experience of parenting.

Parents who were able to adapt their expectations for their parenting and their life—often through an acceptance of their child's Autistic identity—spoke of experiencing parenting positively. While many parents—both Autistic and non-Autistic—spoke of the reality of their parenting not matching their expectations, parents who were able to adjust their expectations in light of their Autistic child's needs (e.g., through increased collaborative parenting) seemed not to resent or blame their child and their Autistic identity. Flexibly responding to the unique needs of their child, regardless of preconceived notions of what “good parenting” might look like, and co-creating a life with their child was, for many parents in this study, a vital step to experiencing their parenting journey positively. Given these results, we suggest that incorporating targeted ways to help parents to reframe and reset their parenting expectations may be beneficial within post-diagnostic supports aimed at parents.

Finally, crucial to parents’ positive experiences was their feelings of self-efficacy, as attested in other literature (e.g., Almendingen & Pilkington, 2023; Brennan et al., 2024). Here, many parents, regardless of neurotype, found joy in how parenting made them feel about themselves, and their joy was often intimately connected to their child's happiness and to their experience of self-efficacy and self-growth. Indeed, the more a parent experienced their child's happiness, the more they felt able to be present and connect with their child, and the more secure they felt in their confidence to parent their child, which in turn fed their child's happiness. Equally, however, parents could find connection through shared negative experience with their child, especially when their child needed them, and they felt they could provide safety, help or support. Parents in this study thus spoke about needing to be needed, as well as needing to feel as though they could provide safety and meet their child's needs. It is important that future programs and research into parental and familial support recognize this need to refocus efforts to actively developing happiness, rather than simply mitigating stressors.

### Limitations

This study has several limitations. First, the findings may not be representative of the diversity of parents of Autistic children. The sample, as well as predominantly being well-educated and White, were primarily mothers, and the experiences of culturally diverse people and of fathers and nonbinary parents were underrepresented, as is usual in studies such as these (Thom-Jones et al., 2024). We did not explicitly ask about the influence of cultural expectations or gender on parents’ experiences, so we were not able to assess the impact of these on parenting experiences. Equally, most (although not all) of our participants had the financial means and educational background to access learning about autism, which may not be representative of those parents without such means. Similarly, since interviews were conducted online, those parents with higher support or communication needs, and those who were financially disadvantaged, may have been excluded. Second, all Autistic parents were late-identified, and their experiences may not represent those of Autistic parents who were identified in childhood, or at least before their child was diagnosed. Third, only 11% of participants were parents of nonspeaking or minimally speaking children, or children with intellectual disability; given that current estimates suggest that around one-third of Autistic children are nonspeaking (Jaswal et al., 2024), this demographic was significantly underrepresented in this study. This cohort is routinely excluded from research (Tager-Flusberg & Kasari, 2013), although it was encouraging that our findings from the few parents of nonspeaking Autistic children echoed those of speaking Autistic children. Fourth, because we spoke to parents of primary school-aged children, we did not capture potential additional complexities of, for example, parenting adolescents, which would undoubtedly affect parents’ experiences, and is an important area for future research. Finally, because recruitment occurred at least somewhat through community networks, there is also a possibility that participants were skewed to a neuro-affirming mindset, or that prospective participants chose not to take part because of this perception. Nevertheless, many parents came to the study without prior knowledge and parents spoke frankly about their experiences, including about challenges and difficulties.

## Conclusion

Our study shows that relationships between parents—regardless of their neurotype—and their Autistic children are complex and challenging but can be characterized by reciprocated connection and love. While further research into what factors actively promote positive, joyful, and fulfilling parenting journeys is warranted, these findings preliminarily suggest that acceptance, flexibility, and the reframing of expectations are all conduits to parenting self-efficacy and joy for parents of Autistic children. As McMahon (2022) points out, researchers rarely attend to “capturing the benefits [of parenting]” because doing so “is not easy: they are elusive, conditional, abstract, may take years to be realized, and difficult to quantify and measure” (p. 9). Yet, this study shows that parenting Autistic children—although frequently described as having primarily negative parental outcomes—brings significant positive experiences and rewards to parents, regardless of their neurotype. Future autism research needs to attend to the full complexity of parents’ experiences as they parent their Autistic children.

## Supplemental Material

sj-docx-1-dli-10.1177_23969415251357222 - Supplemental material for “There is Nowhere Else That I’d Rather be Than with Them”: Parents’ Positive Experiences Parenting Autistic Children Supplemental material, sj-docx-1-dli-10.1177_23969415251357222 for “There is Nowhere Else That I’d Rather be Than with Them”: Parents’ Positive Experiences Parenting Autistic Children by Melanie Heyworth, Catherine McMahon, Diana Weiting Tan and Elizabeth Pellicano in Autism & Developmental Language Impairments

## Notes

1. We have chosen to capitalize the word Autistic to indicate a proper adjective, which reflects its status as a robust and valued identity and shared community, similar to the Deaf community.

2. The NDIS, funded by the Australian Government, provides no-fault insurance cover for Australians (aged <65 years), who are born with or acquire a permanent and significant disability. It provides disability funding for support and services directly to individuals and is designed to give them more choice and control over their care.
