# Supplementary material for: “There is Nowhere Else That I’d Rather be Than with Them”: Parents’ Positive Experiences Parenting Autistic Children
Source: Autism Dev Lang Impair. 2025 Jul 6;10:23969415251357222. doi: 10.1177/23969415251357222 (PMC12284749; doi:10.1177/23969415251357222)
Supplement: sj-docx-1-dli-10.1177_23969415251357222 - Supplemental material for “There is Nowhere Else That I’d Rather be Than with Them”: Parents’ Positive Experiences Parenting Autistic Children [file sj-docx-1-dli-10.1177_23969415251357222.docx]

**Supplementary Materials T1. Full participant characteristics**

| Participant characteristics | Parents of Autistic children (n=80) | | |
| --- | --- | --- | --- |
|  | All parents (n=80) | Autistic parents (n=40)^a^ | Non-autistic parents (n=40)^b^ |
|  | Mean (SD), Range, or N (%)^c^ | | |
| Age (years) | 42 (5.2),  26.5-55.3 | 42.2 (5),  31.9-55.3 | 41.8 (5.4),  26.5-52.2 |
| Age at Autism diagnosis (years)^d^ | NA | 39.6 (5.5),  29-54 | NA |
| Interview duration (min)^e^ | 85 (21.1),  43-195 | 80 (17.6),  43-120 | 89.9 (22.9),  52-195 |
| Gender |  |  |  |
| Woman | 70 (87.5%) | 35 (87.5%) | 35 (87.5%) |
| Man | 7 (8.8%) | 2 (5%) | 5 (12.5%) |
| Non-binary | 3 (3.8%) | 3 (7.5%) | 0 (0%) |
| Transgender | 3 (3.8%) | 3 (7.5%) | 0 (0%) |
| Participants’ country of birth |  |  |  |
| Australia | 63 (78.8%) | 30 (75%) | 33 (82.5%) |
| Cayman Islands | 1 (1.3%) | 0 (0%) | 1 (2.5%) |
| Colombia | 1 (1.3%) | 1 (2.5%) | 0 (0%) |
| China | 2 (2.5%) | 1 (2.5%) | 1 (2.5%) |
| Germany | 1 (1.3%) | 1 (2.5%) | 0 (0%) |
| Ireland | 1 (1.3%) | 0 (0%) | 1 (2.5%) |
| Singapore | 1 (1.3%) | 1 (2.5%) | 0 (0%) |
| South Africa | 1 (1.3%) | 0 (0%) | 1 (2.5%) |
| Tanzania | 1 (1.3%) | 1 (2.5%) | 0 (0%) |
| United Kingdom | 5 (6.3%) | 3 (7.5%) | 2 (5%) |
| United States | 3 (3.8%) | 2 (5%) | 1 (2.5%) |
| Parents’ country of birth^f^ |  |  |  |
| Australia | 90 (56.3%) | 42 (52.5%) | 48 (60%) |
| Austria | 2 (1.3%) | 2 (2.5%) | 0 (0%) |
| Canada | 2 (1.3%) | 2 (2.5%) | 0 (0%) |
| China | 4 (2.5%) | 2 (2.5%) | 2 (2.5%) |
| Colombia | 2 (1.3%) | 2 (2.5%) | 0 (0%) |
| Dutch Indies | 2 (1.3%) | 0 (0%) | 2 (2.5%) |
| Germany | 2 (1.3%) | 2 (2.5%) | 0 (0%) |
| India | 1 (0.6%) | 0 (0%) | 1 (1.25%) |
| Ireland | 2 (1.3%) | 0 (0%) | 2 (2.5%) |
| Italy | 2 (1.3%) | 0 (0%) | 2 (2.5%) |
| Japan | 2 (1.3%) | 0 (0%) | 2 (2.5%) |
| Kenya | 2 (1.3%) | 2 (2.5%) | 0 (0%) |
| Lebanon | 2 (1.3%) | 0 (0%) | 2 (2.5%) |
| Macedonia | 4 (2.5%) | 0 (0%) | 4 (5%) |
| Malta | 2 (1.3%) | 2 (2.5%) | 0 (0%) |
| New Zealand | 4 (2.5%) | 2 (2.5%) | 2 (2.5%) |
| Singapore | 2 (1.3%) | 0 (0%) | 2 (2.5%) |
| South Africa | 5 (3.1%) | 2 (2.5%) | 3 (3.75%) |
| Thailand | 2 (1.3%) | 2 (2.5%) | 0 (0%) |
| United Kingdom | 18 (11.3%) | 10 (12.5%) | 8 (10%) |
| United States | 8 (5%) | 6 (7.5%) | 2 (2.5%) |
| Aboriginal or Torres Strait Islander | 3 (3.8%) | 2 (5%) | 1 (2.5%) |
| State of residence (Australia) |  |  |  |
| ACT | 2 (2.5%) | 1 (2.5%) | 1 (2.5%) |
| NSW | 22 (27.5%) | 7 (17.5%) | 15 (37.5%) |
| QLD | 12 (15%) | 8 (20%) | 4 (10%) |
| SA | 6 (7.5%) | 6 (15%) | 0 (0%) |
| VIC | 22 (27.5%) | 10 (25%) | 12 (30%) |
| WA | 16 (20%) | 8 (20%) | 8 (20%) |
| MMM status^g^ |  |  |  |
| 1 (Metropolitan) | 61 (76.3%) | 30 (75%) | 31 (77.5%) |
| 2 (Regional) | 12 (15%) | 5 (12.5%) | 7 (17.5%) |
| 3 (Large rural town) | 2 (2.5%) | 1 (2.5%) | 1 (2.5%) |
| 4 (Medium rural town) | 0 (0%) | 0 (0%) | 0 (0%) |
| 5 (Small rural town) | 5 (6.3%) | 4 (10%) | 1 (2.5%) |
| Parenting arrangements |  |  |  |
| Co-parenting | 40 (50%) | 19 (47.5%) | 21 (52.5%) |
| Primary caregiver | 30 (37.5%) | 15 (37.5%) | 15 (37.5%) |
| Solo parenting | 10 (12.5) | 6 (15%) | 4 (10%) |
| Educational attainment |  |  |  |
| High school | 3 (3.8%) | 2 (5%) | 1 (2.5%) |
| TAFE certificate or diploma | 17 (21.3%) | 9 (22.5%) | 8 (20%) |
| Trade/technical certificate | 1 (1.3%) | 1 (2.5%) | 0 (0%) |
| University qualification | 57 (71.3%) | 27 (67.5%) | 30 (75%) |
| Other | 2 (2.5%) | 1 (2.5%) | 1 (2.5%) |
| Current employment status |  |  |  |
| Disability benefits | 1 (1.3%) | 0 (0%) | 1 (2.5%) |
| Employed full-time | 24 (30%) | 8 (20%) | 16 (40%) |
| Employed part-time | 22 (27.5%) | 9 (22.5%) | 13 (32.5%) |
| Homemaker | 16 (20%) | 11 (27.5%) | 5 (12.5%) |
| Seeking opportunities | 1 (1.3%) | 1 (2.5%) | 0 (0%) |
| Self-employed | 7 (8.8%) | 4 (10%) | 3 (7.5%) |
| Studying | 6 (7.5%) | 4 (10%) | 2 (5%) |
| Other | 2 (2.5%) | 2 (5%) | 0 (0%) |
| Undisclosed | 1 (1.3%) | 1 (2.5%) | 0 (0%) |
| Co-occurring conditions |  |  |  |
| Attention-Deficit Hyperactivity Disorder (ADHD) | 25 (31.5%) | 19 (47.5%) | 6 (15%) |
| Anxiety | 38 (47.5%) | 23 (57.5%) | 15 (37.5%) |
| Bipolar | 1 (1.3%) | 1 (2.5%) | 0 (0%) |
| Chronic Fatigue | 5 (6.3%) | 3 (7.5%) | 2 (5%) |
| Depression | 28 (35%) | 17 (42.5%) | 11 (27.5%) |
| Drug / alcohol dependence | 5 (6.3%) | 3 (7.5%) | 2 (5%) |
| Dyslexia | 3 (3.8%) | 3 (7.5%) | 0 (0%) |
| Eating disorder | 5 (6.3%) | 4 (10%) | 1 (2.5%) |
| Obsessive Compulsive Disorder (OCD) | 3 (3.8%) | 3 (7.5%) | 0 (0%) |
| Post-Traumatic Stress Disorder (PTSD) | 16 (20%) | 11 (27.5%) | 5 (12.5%) |
| Other | 9 (11.3%) | 6 (15%) | 3 (7.5%) |
| Undisclosed | 7 (8.8%) | 2 (5%) | 5 (12.5%) |
| National Disability Insurance Scheme (NDIS) plan^h^ |  |  |  |
| No | NA | 29 (72.5%) | NA |
| Yes | NA | 9 (22.5%) | NA |
| Pending application | NA | 2 (5%) | NA |

^a^ Two Autistic parents chose “I am questioning my Autistic identity” in the demographic survey; since these participants consistently identified themselves as Autistic in the interview and in email correspondence, they have been included in the Autistic parent cohort.

^b^ Four non-autistic parents chose “I am questioning my Autistic identity” in the demographic survey; since these participants consistently identified themselves as non-autistic in the interview and in email correspondence, they have been included in the non-autistic parent cohort.

^c^ Data are mean (SD; range) or n (%). Percentages may not sum to 100% due to rounding issues. NA = not applicable.

^d^ Age at formal diagnosis, or age at which participants began to self-identify.

^e^ Five participants (three Autistic and two non-autistic) chose to complete their interviews in writing.

^f^ Participants reported on both of their parents; all participants’ parents n=160.

^g^ Classifications are based on the Modified Monash Model (2019), which defines whether an Australian location is metropolitan, rural or remote. No participants reported living in remote or very remote areas.

^h^ NDIS plan listing Autism as the primary disability.

**Supplementary Materials T2. Full Autistic child characteristics, as reported by parents**

| Child characteristics | All parent participants (n=80) | Autistic parent participants (n=40) | Non-autistic parent participants (n=40) |
| --- | --- | --- | --- |
|  | Mean (SD), Range, or N (%)^a^ | | |
| Total number of Autistic children | n=87 | n=47 | n=40 |
| Age (years) | 10 (1.7),  6.3-12.9 | 10.08 (1.7),  7.3-12.8 | 10 (1.8),  6.3-12.9 |
| Diagnosis |  |  |  |
| Autism Spectrum Disorder | 85 (97.7%) | 46 (97.9%) | 39 (97.5%) |
| Autistic Disorder | 1 (1.2%) | 1 (2.1%) | 0 (0%) |
| Asperger’s Syndrome | 1 (1.2%) | 0 (0%) | 1 (2.5%) |
| Age at Autism diagnosis (years) | 6.38 (2.6),  1.1-11.8 | 6.22 (2.5),  1.1-11.3 | 6.56 (2.8),  2-11.8 |
| Gender |  |  |  |
| Girl | 35 (40.2%) | 20 (42.6%) | 15 (37.5%) |
| Boy | 50 (57.5%) | 25 (53.2%) | 25 (62.5%) |
| Non-binary | 2 (2.3%) | 2 (4.3%) | 0 (0%) |
| Communication |  |  |  |
| Speaking | 83 (95.4%)^b^ | 46 (97.9%)^c^ | 37 (92.5%)^d^ |
| Non-speaking | 4 (4.6%) | 1 (2.1%) | 3 (7.5%) |
| Current educational setting |  |  |  |
| Autism or disability specific school | 4 (4.6%) | 2 (4.3%) | 2 (5%) |
| Distance education | 1 (1.2%) | 0 (0%) | 1 (2.5%) |
| Homeschooling | 13 (14.9%) | 11 (23.4%) | 2 (5%) |
| Mainstream with support | 36 (41.4%) | 17 (36.2%) | 19 (47.5%) |
| Mainstream without support | 20 (23%) | 9 (19.2%) | 11 (27.5%) |
| Specialist school | 1 (1.2%) | 0 (0%) | 1 (2.5%) |
| Support unit within mainstream | 5 (5.8%) | 2 (4.3%) | 3 (7.5%) |
| Unschooling | 7 (8.1%) | 6 (12.8%) | 1 (2.5%) |
| Co-occurring conditions |  |  |  |
| ADHD | 55 (63.2%) | 29 (61.7%) | 26 (65%) |
| Anxiety | 47 (54%) | 27 (57.5%) | 20 (50%) |
| Cerebral Palsy | 1 (1.2%) | 0 (0%) | 1 (2.5%) |
| Depression | 4 (4.6%) | 2 (4.3%) | 2 (5%) |
| Dyscalculia | 2 (2.3%) | 1 (2.1%) | 1 (2.5%) |
| Dysgraphia | 5 (5.8%) | 3 (6.4%) | 2 (5%) |
| Dyslexia | 9 (10.3%) | 7 (14.9%) | 2 (5%) |
| Dyspraxia | 4 (4.6%) | 3 (6.4%) | 1 (2.5%) |
| Eating disorder | 3 (3.5%) | 3 (6.4%) | 0 (0%) |
| Ehlers Danlos or Hypermobility Syndromes | 2 (2.3%) | 2 (4.3%) | 0 (0%) |
| Epilepsy | 2 (2.3%) | 2 (4.3%) | 0 (0%) |
| Global Development Delay (GDD) | 5 (5.8%) | 1 (2.1%) | 4 (10%) |
| Intellectual Disability | 5 (5.8%) | 2 (4.3%) | 3 (7.5%) |
| OCD | 6 (6.9%) | 4 (8.5%) | 2 (5%) |
| Oppositional Defiance Disorder (ODD) | 3 (3.5%) | 1 (2.1%) | 2 (5%) |
| PTSD | 1 (1.2%) | 1 (2.1%) | 0 (0%) |
| Tourette Syndrome | 1 (1.2%) | 0 (0%) | 1 (2.5%) |
| None | 13 (14.9%) | 7 (14.9%) | 6 (15%) |
| Other | 3 (3.5%) | 2 (4.3%) | 1 (2.5%) |
| NDIS plan^e^ |  |  |  |
| No | 5 (5.8%) | 3 (6.4%) | 2 (5%) |
| Yes | 81 (94.2%) | 44 (93.6%) | 37 (92.5%) |
| Pending application | 1 (1.2%) | 0 (0%) | 1 (2.5%) |

^a^ Data are mean (SD; range) or n (%). Percentages may not sum to 100% due to rounding issues. NA = not applicable.

^b^ Of these, four parents reported that their child had a significant language delay (n=4, 5%).

^c^ Of these, two parents reported that their child had a significant language delay (n=2, 4%).

^d^ Of these, two parents reported that their child had a significant language delay (n=2, 5%).

^e^ NDIS plan listing Autism as the primary disability.

**Supplementary Materials S3. Interview schedule**

**Demographics**

What is your date of birth (DOB)?

What is your postcode?

Are you formally diagnosed as Autistic, self-identifying, or questioning?

At what age did you receive your Autism diagnosis?

What is your Autistic child’s first name?

What is their date of birth?

What is your child’s diagnosis? When did they receive it?

*[When applicable]*

What is your non-autistic child’s first name?

What is their DOB?

**Five-Minute Speech Sample**

Firstly, I’d like to hear your thoughts about [Autistic child] in your own words and without my interrupting you with any questions or comments. When I ask you to begin, I’d like you to speak for 5 minutes. When you’ve spoken for 5 minutes, I’ll let you know. After you have begun to speak, I prefer not to answer any questions. So, I’d like you to tell me what kind of a person [Autistic child] is and how the two of you get along together. Are there any questions you would like to ask me before we begin?

*[When applicable]*

Now, I’m going to ask the same question about [non-autistic child]. I’d like to hear your thoughts about [non-autistic child] in your own words and without my interrupting you with any questions or comments. When I ask you to begin, I’d like you to speak for 5 minutes. When you’ve spoken for 5 minutes, I’ll let you know. After you have begun to speak, I prefer not to answer any questions. So, I’d like you to tell me what kind of a person [non-autistic child] is and how the two of you get along together. Are there any questions you would like to ask me before we begin?

*[Written version]*

Firstly, I’d like to hear your thoughts about [Autistic child] in your own words. This is meant to be a spontaneous response, so it doesn’t need to be polished or edited in any way. When you begin writing, I’d like you to write around 650-750 words. When a participant responds to this question in speech, I try not to answer any questions or interrupt them, so I would love you to try and write your answer just to this question in one sitting, and without further clarification from me if that is feasible.

So, I’d like you to tell me what kind of a person [Autistic child] is and how the two of you get along together.

**Semi-structured interview**

1a) Tell me more about your experiences of parenting [Autistic child].

Prompts:

- What are some of the fulfilling or joyful parts of parenting [Autistic child]?
- What are some of the challenging parts of parenting [Autistic child]?
- What are some of your day-to-day or week-to-week routines that seem to shape the rhythm of your time as a parent?
- What sorts of things do you do with [Autistic child]?

*[When applicable]*

1b) Are there differences in your experiences in parenting [Autistic child] and [non-autistic child]?

Prompts:

- If you reflected on your experiences of parenting [Autistic child] and [non-autistic child], are the parts that are fulfilling or challenging different? If so, how?
- Are the routines you have with [non-autistic child] different to those you have established with [Autistic child]? What is the effect or impact of those differences?
- What sorts of things do you do with [non-autistic child]? Are these different to [Autistic child]? If yes, why? What is the effect or impact of those differences?

2a) How would you describe your attachment – or your connection – with [Autistic child]?

Prompts:

- Are there moments where you feel a deep connection with [Autistic child]? What are they?
- Are there times when you feel disconnected from [Autistic child]? Tell me about them.
- What emotions do you feel about [Autistic child] or how do they make you feel?
- Do you think [Autistic child] reciprocates these feelings? What indicates that?

*[When applicable]*

2b) Any there any differences in your feelings of attachment and connection with [non-autistic child]?

Prompts:

- Are there moments where you feel a deep connection with [non-autistic child]? What are they?
- Are there times when you feel disconnected from [non-autistic child]? Tell me about them.
- What emotions do you feel about [non-autistic child] or how do they make you feel?
- Do you think [non-autistic child] reciprocates these feelings? What indicates that?

2c) What is the thing that, in your opinion, most characterises the relationship between you and [Autistic child]?

What are your expectations for the future regarding your relationship with [Autistic child]?

*[When applicable]*

2d) What is the thing that, in your opinion, most characterises the relationship between you and [non-autistic child]?

What are your expectations for the future regarding your relationship with [non-autistic child]?

3) Tell me about how good a job you feel you are doing as a parent.

Prompts:

- Do you think you do a good job parenting? What areas do you think you do really well in?
- Are there times you don’t think you’re a very good parent? Tell me about them.
- Do you think you do as good a job parenting your different children? What elements play into how well you think you’re doing?

4) Tell me a bit about when your child experiences “big emotions”; how do you respond as a parent?

Prompts:

- *[When applicable]* Are your parenting responses the same for all your children? Why/why not?
- What factors play into your response to your child/ren’s “big emotions”?
- What do you think about your child/ren when they are experiencing “big emotions”? *[When applicable]* Is this different for your different children?

5) Do you feel you understand why your child/ren behave the way they do?

Prompts:

- Do you think you understand the motivations underlying your child/ren’s behaviours?
- *[When applicable]* Is this the same for all your children?
- What helps you to understand your child/ren’s motivations?

6) What are your past experiences of therapies and interventions?

Prompts:

- Did any past therapies or interventions focus on your parenting? How did that make you feel? Do you think they worked?
- How do you think these have influenced your relationship with your child?

7) Remembering the time your Autistic child was diagnosed Autistic, what information or support do you wish you had been given then, to help you in your parenting journey?

Is there anything else you would like to add, or want me to know, that you haven’t had the opportunity to talk about?

**End of formal questions.**
